# Contraception and Healthcare Utilization by Reproductive-Age Women Who Use Drugs in Rural Communities: a Cross-Sectional Survey

**DOI:** 10.1007/s11606-022-07558-6

**Published:** 2022-06-15

**Authors:** Ximena A. Levander, Canyon A. Foot, Sara L. Magnusson, Ryan R. Cook, Jerel M. Ezell, Judith Feinberg, Vivian F. Go, Kathryn E. Lancaster, Elizabeth Salisbury-Afshar, Gordon S. Smith, Ryan P. Westergaard, April M. Young, Judith I. Tsui, P. Todd Korthuis

**Affiliations:** 1 Division of General Internal Medicine & Geriatrics, Addiction Medicine Section, Department of Medicine, Oregon Health & Science University, Portland, OR USA; 2 Research & Evaluation, Comagine Health, Portland, OR USA; 3 Africana Studies and Research Center, Cornell Center for Health Equity, Cornell University, Ithaca, NY USA; 4 Department of Behavioral Medicine and Psychiatry, West Virginia University, Morgantown, WV USA; 5 Department of Medicine Section of Infectious Diseases, West Virginia University, Morgantown, WV USA; 6 Department of Health Behavior, School of Global Public Health, University of North Carolina – Chapel Hill, Chapel Hill, NC USA; 7 Division of Epidemiology, College of Public Health, Ohio State University, Columbus, OH USA; 8 Department of Family Medicine and Community Health, University of Wisconsin, Madison, WI USA; 9 Department of Epidemiology and Biostatistics, School of Public Health, West Virginia University, Morgantown, WV USA; 10 Division of Infectious Diseases, Department of Medicine, School of Medicine and Public Health, University of Wisconsin, Madison, WI USA; 11 Department of Epidemiology, College of Public Health, University of Kentucky, Lexington, KY USA; 12 Center on Drug and Alcohol Research, University of Kentucky, Lexington, KY USA; 13 Department of Medicine, Division of General Internal Medicine, University of Washington, Seattle, WA USA

**Keywords:** opioid-related disorders, women’s health, rural health services, contraception, substance use

## Abstract

**Background:**

Women who use drugs (WWUD) have low rates of contraceptive use and high rates of unintended pregnancy. Drug use is common among women in rural U.S. communities, with limited data on how they utilize reproductive, substance use disorder (SUD), and healthcare services.

**Objective:**

We determined contraceptive use prevalence among WWUD in rural communities then compared estimates to women from similar rural areas. We investigated characteristics of those using contraceptives, and associations between contraceptive use and SUD treatment, healthcare utilization, and substance use.

**Design:**

Rural Opioids Initiative (ROI) — cross-sectional survey using respondent-driven sampling (RDS) involving eight rural U.S. regions (January 2018–March 2020); National Survey on Family Growth (NSFG) — nationally-representative U.S. household reproductive health survey (2017–2019).

**Participants:**

Women aged 18–49 with prior 30-day non-prescribed opioid and/or non-opioid injection drug use; fecundity determined by self-reported survey responses.

**Main Measures:**

Unweighted and RDS-weighted prevalence estimates of medical/procedural contraceptive use; chi-squared tests and multi-level linear regressions to test associations.

**Key Results:**

Of 855 women in the ROI, 36.8% (95% CI 33.7–40.1, unweighted) and 38.6% (95% CI 30.7–47.2, weighted) reported contraceptive use, compared to 66% of rural women in the NSFG sample. Among the ROI women, 27% had received prior 30-day SUD treatment via outpatient counseling or inpatient program and these women had increased odds of contraceptive use (aOR 1.50 [95% CI 1.08–2.06]). There was a positive association between contraception use and recent medications for opioid use disorder (aOR 1.34 [95% CI 0.95–1.88]) and prior 6-month primary care utilization (aOR 1.32 [95% CI 0.96–1.82]) that did not meet the threshold for statistical significance.

**Conclusion:**

WWUD in rural areas reported low contraceptive use; those who recently received SUD treatment had greater odds of contraceptive use. Improvements are needed in expanding reproductive and preventive health within SUD treatment and primary care services in rural communities.

## INTRODUCTION

In the United States (U.S.), women are using drugs at increasing rates,^1^ with consequent rises in overdose deaths, substance-exposed pregnancies, and other related consequences over the past two decades.^1,2^ The national prevalence of maternal opioid-related diagnoses at time of hospital delivery^3,4^ and neonatal opioid withdrawal syndrome (NOWS) have increased considerably,^4^ with trends indicating higher rates of maternal opioid-related diagnoses and NOWS in rural compared to urban U.S. areas.^5^ Reproductive-age women who use drugs (WWUD) experience unintended pregnancies at up to double the rate than the general population.^6^ A contributing factor is substantially lower rates of highly effective contraceptive utilization among WWUD as compared to women who do not use drugs.^7^ Nationwide household surveys including the National Survey on Family Growth (NSFG), which include no questions about substance use, likely fail to capture the reproductive health needs of WWUD, particularly those in rural communities.^8^ Despite increasing awareness surrounding unintended pregnancies and associated maternal and neonatal morbidity and mortality among WWUD, little is known about disparities in utilization of reproductive health services of WWUD in rural communities.

Individual, community, and environmental factors contribute to 60–90% of pregnancies being unintended among WWUD,^6^ compared to 45% among the general U.S. population.^9^ WWUD less often use highly effective contraceptive methods (long-acting reversible contraception — intrauterine device or implant), and effective methods (hormonal pill, patch, injection, or ring),^7,10,11^ while relying heavily on condoms for contraception.^7,12^ Condoms are less effective at preventing pregnancy, given imperfect and inconsistent use. Contraceptive use and reproductive choice by WWUD is further influenced by high rates of intimate partner violence and sexual and reproductive coercion.^13,14^ While not all WWUD want or need highly or moderately effective contraception, the notable differences in unintended pregnancy rates may indicate a gap in reproductive health access and utilization and necessitates further investigation and interventions. Pregnant and parenting WWUD also face considerable stigma,^15^ frequently delaying or avoiding prenatal care, citing concerns around child protective service involvement and potential custodial loss.^16,17^ Delays to care contribute to maternal and neonatal complications including placental rupture, premature delivery, NOWS, and intrauterine growth restriction.^18^

Gender and sex-related factors unique to women contribute to their initial substance use, progression from initial use to SUD, and substance use disorder (SUD) treatment engagement and retention.^2^ WWUD tend to engage in higher-risk injection and sexual behaviors, experience higher rates of intimate partner violence, and have higher risk of acquiring HIV and HCV than their male counterparts.^19–21^ WWUD from rural communities experience additional layers of risk compared to WWUD in urban settings.^22,23^ In rural communities, WWUD experience poorer health outcomes partially due to unique barriers to accessing healthcare and reproductive services (limited service availability, insurance/financial concerns, limited transportation),^22,24,25^ and SUD treatment (specialist shortages,^26^ drive times to specialists,^27^ and rural-specific stigma^28,29^).

Large-scale efforts are needed to expand access to family planning and reproductive services for WWUD in various care settings, particularly in rural communities where these services are generally lacking.^30^ However, there are limited data on current contraceptive use in the rural U.S. by WWUD. We sought to estimate contraceptive use prevalence among WWUD in rural communities compared to a national sample of women from rural areas, one of the first studies to do so. We hypothesized that WWUD in rural communities would have lower prevalence of contraceptive use compared to a nationally representative sample of women living in rural areas. We also aimed to identify characteristics of WWUD in rural communities associated with contraceptive use and to evaluate possible associations between contraceptive use and SUD treatment utilization, health care utilization, substance use, and HIV/HCV testing. We hypothesized that recent SUD treatment and healthcare utilization would be associated with increased contraceptive utilization, those with recent substance use would have reduced contraceptive utilization, and recent HIV/HCV testing would be associated with increased contraceptive utilization.

## METHODS

### Rural Opioids Initiative — Study Design, Participants, and Data Collection

This investigation is a sub-analysis of the Rural Opioids Initiative (ROI) — a multi-site cross-sectional survey of people who use drugs from ten U.S. states (Illinois; Kentucky; North Carolina; New England which included Massachusetts, Vermont, and New Hampshire; Ohio; Oregon; Wisconsin; and West Virginia) with recruitment from January 2018 to March 2020.^31^ The ROI collected data on demographics, drug use, consequences of use, SUD treatment, HIV/HCV screening and treatment, and healthcare utilization. Eligible participants reported use of any opioid via any administration route and/or any other drug via injection in the prior 30 days “to get high” (e.g., smoking heroin; injecting heroin and smoking cocaine; injecting methamphetamine) except for Wisconsin which limited to injection use only. Inclusion criterion for all sites was age ≥18 years old except two states (Illinois, Wisconsin) where the age criterion was ≥15 years old. For this analysis, our study population included all women 18 to 49 years old who were able to become pregnant determined by survey responses. The upper age limit of 49 years aligns with the NSFG. The NSFG includes women between 15 and 18 years old; however, given only two ROI sites included this younger age range, with only two participants otherwise meeting inclusion criteria, they were excluded from further analysis.

All sites conducted recruitment using modified chain-referral sampling,^31,32^ a strategy based on respondent-driven sampling (RDS) methods to improve sampling of “hidden” populations.^33,34^ Study sites identified “seed” participants who represented local population demographics and recruited within their network. Participants were linked via referral chains when estimating weighted prevalence rates. Additional ROI data collection and management details are previously published.^31^ The ROI data coordinating center collected, standardized, managed, and distributed data for analyses approved by the publication committee. All study procedures were approved by the Institutional Review Board at each site.

### National Survey of Family Growth

The National Center for Health Statistics conducts the NSFG,^35,36^ collecting nationally representative household estimates of family planning and reproductive health topics for men and women.^35^ The NSFG does not ask about drug use and as a household survey does not include those currently incarcerated or unhoused.^8^ The NSFG age limit expanded from 15–44 to 15–49 years old with the 2015–2017 cohort, and purposefully oversamples non-Hispanic Blacks, Hispanics, and teens.^35,36^ The NSFG uses U.S. Census Bureau Office of Management and Budget for residence location. We included NSFG respondents residing outside of U.S. Census Metropolitan Statistical Areas to approximate ROI regions.

### Measures

We determined the ROI population of reproductive-age women who were not currently pregnant but could become pregnant via survey responses. The ROI survey asked all participants their gender (male, female, transgender, other) and was designed so only participants identifying as “female” were asked their pregnancy status (yes, no, or don’t know). Participants who selected “no” or “don’t know” regarding pregnancy status were then asked, “Are you using any medical forms of birth control such as pills, an IUD, implant, injection, ring, or patch, or are your ‘tubes tied’?” (yes, no, not applicable [no vaginal sex with a male in past 10 months], not applicable [not physically able to get pregnant right now — hysterectomy, health condition, menopause], or decline to answer). Those who answered “yes” or “no” were included in the analysis. We use the term “medical/procedural contraceptive” to encompass all medical forms of hormonal contraception (pill, injection, ring, patch) and all procedural/surgical contraceptive measures (intrauterine device, implant, tubal ligation) consistent with the ROI survey. Participants were not asked additional details about contraceptive methods, family planning preferences, or adequate information to assess use of condoms or other barrier methods as contraception.

The primary variable was medical/procedural contraceptive use. Characteristics potentially associated with contraceptive use were examined on the basis of previous literature and a priori hypotheses.^9,37,38^ We included categorical characteristics: age; education level; race/ethnicity; relationship status. We included yes/no characteristics: homelessness in prior 6 months; incarceration in prior 6 months; trading sex for drugs, money, or housing in prior 30 days; and having had sex without a condom ≥ 1 time in prior 30 days.

We divided SUD treatment service utilization within the prior 30 days into two categories: (1) medication for opioid use disorder (MOUD) use (buprenorphine, methadone, or naltrexone), and (2) SUD treatment via outpatient counseling or inpatient/residential program. Survey participants could indicate use of more than one SUD treatment (e.g., prescribed buprenorphine and engaged in counseling). Another outcome was healthcare location in the prior 6 months with four categories: (1) ambulatory care (private clinician or community health), (2) acute care (urgent care or emergency department), (3) health department or mobile van, (4) no care received. Other outcomes included the following: prior 30-day substance use (opioids, methamphetamine, cocaine, alcohol, or tobacco) and prior year HIV/HCV testing.

### Analyses

We used descriptive statistics to summarize participant characteristics, prevalence estimates, and site-specific variation of medical/procedural contraceptive use. Overall, unweighted and RDS-weighted estimates of medical/procedural contraceptive use and 95% confidence intervals were calculated using mixed-effects models.^34^ Study site was the random effect in these models, and estimates were obtained by averaging over site-specific effects. We then stratified the data by site to compute site-specific estimates of medical/procedural contraceptive use and bootstrapped 95% confidence intervals. West Virginia was excluded from weighted estimates due to incomplete RDS data at time of analysis.

To compare rates of medical/procedural contraceptive use between the ROI and NSFG groups, we restricted the NSFG sample of women to match the ROI inclusion criteria, excluding currently pregnant NSFG respondents, those not sexually active within the past 10 months, and those reporting infertility due to a medical condition or non-contraceptive surgery. Differences in age, race/ethnicity, and education between the ROI and NSFG samples were assessed using chi-squared test with Rao and Scott’s second-order correction to account for weighting in the NSFG survey data. We fit a logistic regression model on the pooled sample and calculated marginal means to estimate the rates of medical/procedural contraceptive use in the ROI and NSFG groups after adjusting for demographic differences.

Within the ROI sample, we assessed bivariate associations between contraceptive use and participant characteristics using Pearson’s chi-square test. Multivariable associations were assessed using generalized linear mixed methods with site-level random intercepts between contraceptive use and MOUD treatment; SUD treatment with outpatient counseling or inpatient program; primary care access with ambulatory services (private clinician or community health center); recent substance use; and HIV/HCV testing. Potential confounders with *p*-values ≤ 0.10 in bivariate analyses were included as covariates in each model, in addition to the main independent variable of medical/procedural contraceptive use. Analyses were conducted in R v.4.0.5 using the ‘lme4’ package.

## RESULTS

The ROI recruited 3,048 participants from eight study sites. Sixteen (0.5%) who self-identified as transgender or other gender and 30 (3.4%) women who were currently pregnant were excluded from the analytic sample. Our sample included 855 WWUD, with average age 33 years (SD 8), who were predominantly White (83%) and insured (79%) (Table 1). Overall, 50% had engaged in condom-less sex in the prior 30 days and 53% had experienced homelessness in the prior 6 months. The overall prevalence of medical/procedural contraceptive use was 36.8% unweighted (95% CI 33.7, 40.1) and 38.6% weighted (95% CI 30.7, 47.2) (Table 2). Overall and site-specific prevalence estimates of medical/procedural contraceptive use are listed in Table 2.

**Table 1.** Characteristics of Women Participating in the Rural Opioids Initiative Who Can Become Pregnant by Self-reported Medical/Procedural Contraceptive Use

**Characteristic**	**Overall** ***N*=855**	**No contraception** ***N*=540**	**Contraception use** ***N*** **=315**	***p*-value**
**Age, mean (SD)**	33 (8)	33 (8)	33 (7)	0.4
**Age range (years)**				0.2
18–24	129 (15%)	81 (15%)	48 (15%)	
25–34	381 (45%)	229 (42%)	152 (48%)	
35–49	345 (40%)	230 (43%)	115 (37%)	
**Race/ethnicity**†				>0.9
Black or African American	15 (1.8%)	10 (1.9%)	5 (1.6%)	
Native American	92 (11%)	60 (11%)	32 (10%)	
Other/mixed race/declined	39 (4.6%)	24 (4.4%)	15 (4.8%)	
White	709 (83%)	446 (83%)	263 (83%)	
Hispanic/Latina	35 (4.1%)	22 (4.1%)	13 (4.1%)	
**Educational level**				0.10
Less than high school	174 (20%)	121 (22%)	53 (17%)	
High school diploma/GED	362 (42%)	228 (42%)	134 (43%)	
Some college or higher	319 (37%)	191 (35%)	128 (41%)	
**Relationship status**				0.8
Married	95 (11%)	63 (12%)	32 (10%)	
Divorced, widowed, separated	239 (29%)	144 (28%)	95 (31%)	
Living with partner	213 (26%)	135 (26%)	78 (25%)	
Never married	286 (34%)	180 (34%)	106 (34%)	
**Has health insurance**	673 (79%)	415 (77%)	258 (82%)	0.082
**Healthcare utilization**‡				0.2
Private doc/community health	370 (44%)	217 (41%)	153 (49%)	
Urgent or emergency care	245 (29%)	161 (30%)	84 (27%)	
County health dept/van	39 (4.6%)	23 (4.3%)	16 (5.1%)	
None	144 (17%)	100 (19%)	44 (14%)	
**Experiencing homelessness**‡	456 (53%)	299 (55%)	157 (50%)	0.12
**History of incarceration**‡	342 (40%)	223 (41%)	119 (38%)	0.3
**Utilizing SUD treatment services**§				
MOUD treatment	186 (22%)	106 (20%)	80 (25%)	0.049*
Counseling/inpatient	233 (27%)	130 (24%)	103 (33%)	0.006*
**Condom-less sex**§	425 (50%)	264 (49%)	161 (51%)	0.12
**Exchanging sex for money, drugs**§	82 (9.6)	50 (9.3%)	32 (10%)	0.7
**Substance use**§				
Opioids/heroin/fentanyl	724 (85%)	460 (85%)	264 (84%)	0.6
Methamphetamine/crystal	628 (73%)	411 (76%)	217 (69%)	0.021*
Cocaine/crack	382 (45%)	239 (44%)	143 (45%)	0.7
Alcohol	407 (48%)	266 (49%)	141 (45%)	0.2
Tobacco	782 (91%)	498 (92%)	284 (90%)	0.3
Opioids and methamphetamine	518 (61%)	344 (64%)	174 (55%)	0.015*
Opioids and cocaine	361 (42%)	226 (42%)	135 (43%)	0.8
**Infectious disease screening**‖				
HCV	265 (35%)	168 (35%)	97 (35%)	>0.9
HIV	285 (35%)	173 (33%)	112 (37%)	0.2

*MOUD*, medications for opioid use disorder; *SUD*, substance use disorder

*Statistically significant at *p*<0.05

†Race/ethnicity are mutually exclusive categories. Hispanic includes everyone who is Hispanic. White and Black race include those who are White or Black and not Hispanic.

‡ Reference period: prior 6 months; § reference period: prior 30 days; ‖ reference period: prior year

**Table 2.** Overall and Site-Specific Prevalence of Contraceptive Use, *N*= 855 Rural Opioid Initiative Women Who Use Drugs Who Can Become Pregnant

Study site	**Unweighted**			**Weighted**		
	Contraception use prevalence	Lower	Upper	Contraceptive use prevalence	Lower	Upper
Overall*	36.8%	33.7%	40.1%	38.6%	30.7%	47.2%
Illinois	31.1%	14.7%	45.5%	61.9%	36.2%	82.9%
Kentucky	29.7%	16.2%	41.3%	34.0%	6.00%	61.1%
North Carolina	42.2%	34.4%	50.0%	35.3%	23.4%	48.4%
New England	40.1%	32.9%	47.3%	41.2%	32.3%	49.3%
Ohio	28.1%	20.5%	36.8%	22.9%	11.9%	39.5%
Oregon	33.1%	21.4%	50.0%	44.2%	24.1%	65.4%
Wisconsin	35.8%	30.5%	41.2%	34.3%	25.3%	41.3%
West Virginia	43.0%	27.9%	55.8%	N/D	N/D	N/D

*Overall weighted prevalence of contraceptive use excludes West Virginia

*N/D*, not determined (*no RDS weighted prevalence available*)

In the prior 30 days, 85% of those in ROI sample had used opioids/heroin/fentanyl and 73% had used methamphetamine/crystal (Table 1). Women reporting not using a medical/procedural contraceptive were more likely to have recently used methamphetamine than those who used contraception (76% vs. 69%; *p*=0.021). This difference was also seen in those who used both opioids and methamphetamine (64% vs. 55%; *p*= 0.015) Women who reported medical/procedural contraceptive use were more likely to have received SUD treatment using MOUD in the prior 30 days (25% vs. 20%; *p*=0.049) and SUD treatment via outpatient counseling, or inpatient program (33% vs. 24%; *p*=0.006) compared to those not using contraceptives.

In analyses adjusted for age, race/ethnicity, education level, relationship status, recent homelessness, and insurance status, those in the ROI sample who used SUD treatment services via outpatient counseling or inpatient program within the prior 30 days were 50% more likely to report contraceptive use compared to those who had not (aOR 1.50 [95% CI 1.08–2.06) (Table 3). There was no evidence supporting an association between contraceptive use and recent opioid/heroin/fentanyl, cocaine/crack, combined opioid and cocaine, alcohol, or tobacco use. However, those with recent methamphetamine use, whether alone or in combination with opioids, were less likely to use medical/procedural contraceptive than those without recent methamphetamine use (aOR 0.72 [95% CI 0.52, 0.99]; aOR 0.71 [95% CI 0.53, 0.95], respectively).

**Table 3.** Adjusted Model Results for Medical/Procedural Contraceptive Use Among Rural Opioid Initiative Women 18–49 Years Old (*N*=855)

	**Medical or procedural contraceptive use**		
	Odds ratio	95% CI	*p*-value
**Utilizing SUD treatment services**†			
MOUD treatment	1.34	0.95, 1.88	0.10
Counseling/inpatient	1.50	1.08, 2.06	0.014*
**Health care via ambulatory services**‡	1.32	0.96, 1.82	0.092
**Substance use**†			
Opioids/heroin/fentanyl	0.87	0.59, 1.30	0.5
Methamphetamine/crystal	0.72	0.52, 0.99	0.046*
Cocaine/crack	1.10	0.83, 1.47	0.5
Alcohol	0.88	0.66, 1.17	0.4
Tobacco	0.82	0.49, 1.35	0.4
Opioids and methamphetamine	0.71	0.53, 0.95	0.023*
Opioids and cocaine	1.10	0.82, 1.47	0.5
**Infectious disease screening**§			
HCV	0.94	0.69, 1.30	0.7
HIV	1.19	0.88, 1.62	0.3

*MOUD*, medications for opioid use disorder; *SUD*, substance use disorder

*Statistically significant to *p*<0.05

†Reference period: prior 30 days; ‡ defined as health care use via primary private doctor or community health clinic in prior 6 months; § reference period: prior year

There were 570 women included in the NSFG cohort; 46 were excluded as they were currently pregnant (7.5%). This cohort sample is representative of 7,035,913 housed women of unknown drug use status who live in rural areas. Women in the ROI compared to the NSFG sample of women had a 69% lower likelihood of medical/procedural contraceptive use (aOR 0.31 [95% CI, 0.25–0.4]) after controlling for age, race/ethnicity, and education. Women in the NSFG sample were more racially and ethnically diverse and more likely to have attained some college education or higher compared to the ROI sample (Table 4).

**Table 4.** Comparison of Rural Opioid Initiative and National Survey of Family Growth Sample Demographics and Contraceptive Use

**Characteristic**	**NSFG** ***N*=7,035,913 (*n*, %)**	**ROI** ***N*** **=855 (*n*, %)**	***p*-value**
**Age range (years)**			0.092
18–24	1,051,470 (15%)	129 (15%)	
25–34	2,629,836 (37%)	381 (45%)	
35–49	3,354,607 (48%)	345 (40%)	
**Race/ethnicity**†			<0.001*
Black or African American	858,038 (12%)	15 (1.8%)	
Other/mixed race/declined	277,346 (3.9%)	108 (13%)	
White	5,177,347 (74%)	697 (82%)	
Hispanic/Latina	723,183 (10%)	35 (4.1%)	
**Educational level**			<0.001*
Less than high school	1,342,975 (19%)	174 (20%)	
High school diploma/GED	1,802,815 (26%)	362 (42%)	
Some college or higher	3,890,123 (55%)	319 (37%)	
**Medical/procedural contraceptive use**	4,636,037 (66%)	315 (37%)	<0.001*

*Statistically significant to *p*<0.05

†Race/ethnicity are mutually exclusive categories. Hispanic includes everyone who is Hispanic. White and Black race include those who are White or Black and not Hispanic

## DISCUSSION

Medical/procedural contraceptive use by reproductive-age WWUD in rural U.S. communities was substantially lower (36.8% unweighted across all sites, 38.6% weighted all sites except West Virginia), compared to women from rural communities surveyed in the NSFG, of whom 66% reported medical/procedural contraceptive use. The NSFG likely includes some WWUD; however, given the high prevalence of recent homelessness (53%) and incarceration (40%) among the ROI WWUD, household-based surveys such as NSFG likely miss the majority of those in this highly stigmatized and marginalized population. Our findings highlight the urgency for better addressing the reproductive health and family planning needs for WWUD in rural America who experience marked socioeconomic consequences and medical complications of unintended pregnancies.

We found a positive association in the ROI with prior 30-day SUD treatment involving outpatient counseling or inpatient program suggesting that these interventions that often involve more time with patients are perhaps more likely to give advice regarding contraception and family planning. While recent MOUD use was greater among women using contraception than those not using contraception (25% vs. 20%), the positive association with MOUD treatment and contraception use did not meet the threshold for statistical significance (aOR 1.34 [95% CI 0.95–1.88]). This discrepancy could perhaps be explained by the high prevalence of methamphetamine use in the ROI population, and current lack of FDA-approved medications to treat stimulant use disorders. Overall, the ROI population had a low utilization of SUD treatment, with 22% recently receiving MOUD and 27% utilizing outpatient counseling, or inpatient program — indicating a clear need to expand access and utilization of SUD treatment in rural communities. SUD treatment encounters are a potential missed opportunity to discuss reproductive health and offer contraceptives to those interested.^39^

Among the ROI population, 44% reported they primarily utilized healthcare via ambulatory services with a positive association between using medical/procedural contraceptive and prior 6-month utilization of ambulatory services that did not meet the threshold for statistical significance (aOR 1.32 [95% CI 0.96–1.82]), after controlling for insurance status. This suggests that factors besides disparities in healthcare access and availability may be contributing to lower contraceptive use among WWUD in rural areas. Outpatient clinicians caring for WWUD are frequently addressing more acute medical and psychosocial issues and they may wait until patients are deemed more stable before discussing family planning.^40^ WWUD, particularly those currently pregnant, face considerable stigma — thus may be reluctant or uncomfortable accessing medical services.^41^ WWUD may also have less trust in the healthcare system,^42^ which, along with child custody and criminal/legal concerns, could hinder counseling on reproductive health. Some women are also equivocal about possibly getting pregnant, and WWUD more frequently report ambivalence around child-bearing or believe they cannot conceive.^43^ Clinicians need to consider what constitutes patient-centered contraceptive counseling and family planning,^44^ particularly given the stigma, discrimination, and historical harms WWUD have experienced and concerns around possible or perceived coercion. There is a critical need for development of women-specific integrated programs offering SUD treatment and/or harm reduction services with reproductive health services,^30,45,46^ in various service delivery models. Pregnant and parenting WWUD may also benefit from these types of care models.^47,48^ Rural communities, with less overall healthcare availability, have further limitations in availability of SUD treatment programs that include wraparound services (behavioral health, social services) and family-specific programs.^49^ This further emphasizes the importance of primary care clinics in delivering SUD treatment and reproductive health services for WWUD in rural areas.

Women with prior 30-day methamphetamine use alone or in combination with opioids had a lower likelihood of using medical/procedural contraceptive compared to those not recently using methamphetamine. Lower contraceptive use in women using methamphetamine is noteworthy given methamphetamine is frequently used for energy and sexual enhancement and is associated with higher-risk sexual behaviors and STI, HIV, and HCV transmission.^50^ Of the 855 ROI participants, 50% had reported prior 30-day condom-less sex. Methamphetamine is also disproportionately used in many rural communities,^51,52^ and 73% of women in our study reported recent use. Research into the most effective contraceptive options and family planning services for women who use methamphetamine is warranted.

HIV/HCV testing within the last year was low, at 35%, regardless of contraceptive use. Women who inject drugs are at higher risk for acquiring HIV, HCV, and other infections, given high rates of needle sharing, high-risk sexual behaviors, transactional sex, and engagement in sex work.^21^ Women who inject drugs and those engaging in transactional sex and sex work should be screened for HIV and offered pre- and post-exposure prophylaxis (PrEP/PEP) to reduce HIV acquisition risk.^53^ Testing and treating reproductive-age WWUD for sexually transmitted and blood-borne infections is important for their health and to reduce the likelihood of vertical transmission if they were to become pregnant.^54^ Efforts are needed to expand adoption of clinical practice guidelines around prescribing PrEP/PEP, screening for infectious disease, and offering highly effective HCV curative treatment for WWUD in rural communities in various treatment settings.

### Limitations and Future Directions

There are limitations to our study. When participants were asked about contraceptive use, one choice included not using contraception as they were “not physically able to get pregnant right now” with reasons including “hysterectomy, health condition, menopause.” Given the incorrect assumption among many WWUD that they are unable to get pregnant,^43,55,56^ our sample may underrepresent the number of reproductive-age women who could become pregnant. The cross-sectional survey design only assessed contraceptive use at one timepoint. We also did not collect more detailed information about specific contraceptive methods thus limiting assessing differences in use of highly versus moderately effective methods or use of combined methods. Further research is needed to study contraceptive and family planning preferences and how to provide patient-centered contraceptive counseling for WWUD.^44^ The survey also did not ask participants’ plans or desire for pregnancy or about their use of condoms or other less effective methods including rhythm or withdrawal as primary or additional form of contraception. The ROI sample also lacked racial/ethnic diversity, a major limitation, given Black, Latina, and multi-racial women face increased rates of reproductive coercion and unintended pregnancy.^57^ They also experience gendered racism and reproductive harms by the healthcare system with ongoing mistrust of contraceptive counseling.^58^ Finally, the survey only asked female-identifying participants to answer questions about contraceptives and pregnancy. We note that female-identified individuals are not the only people who use contraception, need reproductive services, and can get pregnant. Studies are needed on approaches to best serve people of all racial/ethnic and gender identities who use drugs and who can become pregnant.

Some sites had large variation between weighted and unweighted prevalence estimates — particularly Illinois which went from a 31.1 to 61.9% estimated prevalence. This can be explained by very short referral chain lengths in Illinois for women who reported contraceptive use, leading to these participants being weighted more heavily. The smaller the network — the number of community connections a participant has — the more these participants are weighted in statistical calculations to account for the possibility that those with smaller networks (shorter referral chains) represent a more difficult to reach and recruit population when calculating prevalence estimates.

## Conclusion

Despite these limitations, our study provides important insights into the lower prevalence of contraceptive use among WWUD in rural communities compared to a general population of rural women. Interventions that expand access to and improve integration of reproductive health services within SUD treatment, primary care, and harm reduction programs for WWUD are urgently needed in rural areas.

## **Abbreviations**

MOUD: medications for opioid use disorder
NOWS: neonatal opioid withdrawal syndrome
NSFG: National Survey on Family Growth
PEP: post-exposure prophylaxis
PrEP: pre-exposure prophylaxis
RDS: respondent driven sampling
ROI: Rural Opioid Initiative
SUD: substance use disorder
U.S.: United States
WWUD: women who use drugs
